# PLoS Takes a Step Backward

**DOI:** 10.1371/journal.pmed.0020278

**Published:** 2005-08-30

**Authors:** John J Pippin

**Affiliations:** **1**Dallas, TexasUnited States of America

The only people who don't know in 2005 that animal research is irrelevant for human disease are those who don't understand it or those who benefit from it. As a physician, clinical researcher, and former animal researcher, I know that though they are our closest genetic relatives, primates have failed as research models virtually whenever they have been used.

As a partial list of failures, allow me to submit the notorious forced smoking experiments, which allowed cigarettes to be promoted widely for decades; the abject failure of a quarter-century of primate research on AIDS to provide any useful insights; the false leads and dangerous vaccines produced during polio research (verified by Albert Sabin, himself); the failure of primate studies to improve risks for birth defects and premature births; and the failure of monkey studies to identify nonsteroidal anti-inflammatory drug cardiovascular risk [1].

The *PLoS Medicine* editors state in hopeful language that the Lassa fever vaccine was successful in four monkeys, and, thus, is a suitable agent for human study [2]. Recall that VaxGen's AIDS vaccine (AIDSVAX) showed great success in primate studies, but was an abject failure in two human clinical trials, including a trial of over 2,500 injection drug users in Thailand [3] and a multinational trial of over 5,000 high-risk individuals [4].

Consider the fruitless decades-long effort to produce an AIDS vaccine in primates, the failure to produce even a single case of human AIDS in any primate studied, or the failure to identify even one useful AIDS drug from primate studies. Genetic and physiological imperatives dictate that no animal model, even higher primates, gives information applicable to humans. The Human Genome Project [5] tells us that there is sufficient genetic diversity among humans that pharmacogenetic and pharmacogenomic techniques will have an increasing role in overcoming problems related to polymorphisms and other variations. We can't even apply scientific findings uniformly to humans, and *PLoS Medicine* is now promoting monkey research?

I am very disappointed that *PLoS Medicine* has regressed to reporting animal research. It is discouraging that in this era of rapid biomedical advancement, and appropriate relegation of animal research to the historical dustbin, PLoS has chosen to re-introduce an anachronistic, medically discredited, and unethical research tool to its reporting.
